# Brain atlas for glycoprotein hormone receptors at single-transcript level

**DOI:** 10.7554/eLife.79612

**Published:** 2022-09-02

**Authors:** Vitaly Ryu, Anisa Gumerova, Funda Korkmaz, Seong Su Kang, Pavel Katsel, Sari Miyashita, Hasni Kannangara, Liam Cullen, Pokman Chan, TanChun Kuo, Ashley Padilla, Farhath Sultana, Soleil A Wizman, Natan Kramskiy, Samir Zaidi, Se-Min Kim, Maria I New, Clifford J Rosen, Ki A Goosens, Tal Frolinger, Vahram Haroutunian, Keqiang Ye, Daria Lizneva, Terry F Davies, Tony Yuen, Mone Zaidi

**Affiliations:** 1 Center for Translational Medicine and Pharmacology, Icahn School of Medicine at Mount Sinai New York United States; 2 Department of Medicine and of Pharmacological Sciences, Icahn School of Medicine at Mount Sinai New York United States; 3 Department of Pathology, Emory University School of Medicine Atlanta United States; 4 Department of Psychiatry, Icahn School of Medicine at Mount Sinai New York United States; 5 Alamak Biosciences Beverly United States; 6 Memorial Sloan Kettering Cancer Center New York United States; 7 Department of Pediatrics, Icahn School of Medicine at Mount Sinai New York United States; 8 Maine Medical Center Research Institute Scarborough United States; 9 Faculty of Life and Health Sciences, and Brain Cognition and Brain Disease Institute, Shenzhen Institute of Advanced technology, Chinese Academy of Sciences Shenzhen China; Medical College of Georgia at Augusta University United States; Medical College of Georgia at Augusta University United States

**Keywords:** expression profiling, follicle-stimulating hormone, thyroid-stimulating hormone, luteinizing hormone, gonadotropin receptors, Mouse

## Abstract

There is increasing evidence that anterior pituitary hormones, traditionally thought to have unitary functions in regulating single endocrine targets, act on multiple somatic tissues, such as bone, fat, and liver. There is also emerging evidence for anterior pituitary hormone action on brain receptors in mediating central neural and peripheral somatic functions. Here, we have created the most comprehensive neuroanatomical atlas on the expression of TSHR, LHCGR, and FSHR. We have used RNAscope, a technology that allows the detection of mRNA at single-transcript level, together with protein level validation, to document *Tshr* expression in 173 and *Fshr* expression in 353 brain regions, nuclei and subnuclei identified using the *Atlas for the Mouse Brain in Stereotaxic Coordinates*. We also identified *Lhcgr* transcripts in 401 brain regions, nuclei and subnuclei. Complementarily, we used ViewRNA, another single-transcript detection technology, to establish the expression of *FSHR* in human brain samples, where transcripts were co-localized in *MALAT1*-positive neurons. In addition, we show high expression for all three receptors in the ventricular region—with yet unknown functions. Intriguingly, *Tshr* and *Fshr* expression in the ependymal layer of the third ventricle was similar to that of the thyroid follicular cells and testicular Sertoli cells, respectively. In contrast, *Fshr* was localized to NeuN-positive neurons in the granular layer of the dentate gyrus in murine and human brain—both are Alzheimer’s disease-vulnerable regions. Our atlas thus provides a vital resource for scientists to explore the link between the stimulation or inactivation of brain glycoprotein hormone receptors on somatic function. New actionable pathways for human disease may be unmasked through further studies.

## Introduction

There is increasing evidence that pituitary hormones traditionally thought of as ‘pure’ regulators of single physiological processes affect multiple bodily systems, either directly or via actions on brain receptors (Zaidi et al., 2018; Abe et al., 2003). We established, for the first time, a direct action of thyroid-stimulating hormone (TSH) on bone and found that TSH receptor (TSHR) haploinsufficiency causes profound bone loss in mice (Abe et al., 2003). We also found that follicle-stimulating hormone (FSH), *hitherto* thought to solely regulate gonadal function, displayed direct effects on the skeleton to cause bone loss (Sun et al., 2006), and on fat cells, to cause adipogenesis and body fat accumulation (Liu et al., 2017). Likewise, we showed that hormones from the posterior pituitary, namely, oxytocin and vasopressin, displayed direct, but opposing, skeletal actions—effects that may relate to the pathogenesis of bone loss in pregnancy and lactation, and in chronic hyponatremia, respectively (Sun et al., 2019; Sun et al., 2016; Tamma et al., 2009; Tamma et al., 2013). To add to this complexity, and in addition to the poorly recognized ubiquity of pituitary hormone receptors, the ligands themselves, or their variants, are expressed widely. We find the expression of a TSHβ variant (TSHβv) in bone marrow macrophages, while oxytocin is expressed by both osteoblasts and osteoclasts (Colaianni et al., 2011; Colaianni et al., 2012; Baliram et al., 2013; Baliram et al., 2016). These studies have together shifted the paradigm from established unitary functions of pituitary hormones to an evolving array of yet unrecognized roles of physiological and pathophysiological importance.

There is a compelling body of literature to support the expression of oxytocin receptors in various brain regions, and their function in regulating peripheral actions, such as social behavior and satiety (Sun et al., 2019; Bale et al., 2001). However, there is relatively scant information on the expression and, importantly, function of the anterior pituitary glycoprotein hormone family of receptors, namely, FSHR, TSHR, and luteinizing hormone/human chorionic gonadotropin receptor (LHCGR). Discrete sites of the rat, mouse, and human brain express receptors for these hormones, with several studies pointing to their relationship to neural functions, such as cognition, learning, neuronal plasticity, and sensory perception, as well as to neuropsychiatric disorders, including affective disorders and neurodegeneration (Crisanti et al., 2001; Emanuele et al., 1985; Lei et al., 1993; Luan et al., 2020; Bi et al., 2020; Blair et al., 2019; Apaja et al., 2004; Naicker and Naidoo, 2018; Table 1). In the light of such discoveries, the link between the stimulation of these receptors in the brain and the regulation of peripheral physiological processes needs further investigation.

**Table 1.** Known functions of thyroid-stimulating hormone receptor (TSHR), follicle-stimulating hormone receptor (FSHR), and luteinizing hormone/human chorionic gonadotropin receptor (LHCGR) in brain.

Receptor	Species	Brain region	Possible function	Reference
TSHR	Rat	Hypothalamus	Aging	Emanuele et al., 1985
	Mice	Hippocampus	Spatial learning and memory	Luan et al., 2020
	Rat	Hypothalamus, hippocampus, pyriform and postcingulate cortex	Thyroid regulation	Crisanti et al., 2001
	Rat	Hypothalamus	Feeding behavior	Burgos et al., 2016
	Human	Hypothalamus, amygdala, cingulate gyrus, frontal cortex, hippocampus, thalamus	Mood disorders	Naicker and Naidoo, 2018
	Quail	Hypothalamus	Seasonal reproduction	Williams, 2011
FSHR	Yak	Hypothalamus, pineal gland	Follicle growth, maturation, estrus	Huo et al., 2017
	Mice	Hippocampus	Mood regulation	Bi et al., 2020
LHCGR	Rat	Hypothalamus	Aging	Emanuele et al., 1985
	Mice	Hippocampus, cortex	Spatial memory, cognition, plasticity	Blair et al., 2019
	Rat	Hippocampus	Brain metabolism	Liu et al., 2007
	Fish	Hypothalamus	Functional roles	Peng et al., 2018
	Mice	Hippocampus	Promote amyloid-β formation	Lin et al., 2010
	Mice	Cortex	Regulation of neurosteroid production	Apaja et al., 2004
	Mice	Hypothalamus, hippocampus, midbrain, cortex	Regulation of reproductive functions	Hämäläinen et al., 1999
	Yak	Hypothalamus, pineal gland	Follicle growth, maturation, estrus	Huo et al., 2017
	Rat	Hypothalamus, hippocampus, dentate gyrus, cerebellum, brainstem, cortex	Cognitive function (Alzheimer’s disease)	Lei et al., 1993

Here, we use RNAscope—a cutting-edge technology that detects single RNA transcripts—to create the most comprehensive atlas of glycoprotein hormone receptors in mouse brain. This compendium of glycoprotein hormone receptors in concrete brain regions and subregions at a single-transcript level should allow investigators to study both peripheral and central effects of the activation of individual receptors in health and disease. Our identification of brain nuclei with the highest density for each receptor should also create a new way forward in understanding the functional engagement of receptor-bearing nuclei within a large-scale functional network.

## Results

Very little is known about the function(s) of anterior pituitary hormone receptors in the brain, except for isolated studies showing a relationship with cognition and affect (Table 1). We therefore used RNAscope to map the expression of *Tshr*, *Lhcgr,* and *Fshr* in the mouse brain; immunofluorescence and qPCR to provide confirmatory evidence for *Tshr* and *Fshr* expression; and ViewRNA and qPCR to examine for *FSHR* expression in AD-vulnerable regions of the human brain. RNAscope, which allows the detection of single transcripts, uses ~20 pairs of transcript-specific double *Z*-probes to hybridize 10-µm-thick whole-brain sections. Preamplifiers first hybridize to the ~28-bp binding site formed by each double *Z*-probe; amplifiers then bind to the multiple binding sites on each preamplifier; and finally, labeled probes containing a fluorescent molecule bind to multiple sites of each amplifier. RNAscope data was quantified on sections from coded mice. Each section was viewed and analyzed using CaseViewer 2.4 (3DHISTECH, Budapest, Hungary) or QuPath v.0.2.3 (University of Edinburgh, UK). The *Atlas for the Mouse Brain in Stereotaxic Coordinates* (Paxinos and Franklin, 2007) was used to identify every nucleus or subnucleus in which we manually counted *Tshr*, *Lhcgr,* or *Fshr* transcripts in every tenth section using a tag feature. Repeat counting of the same section agreed within <2%. Receptor density was calculated by dividing transcript count by the total area (µm^2^, ImageJ) of each region, nucleus or subnucleus. Photomicrographs were prepared using Photoshop CS5.1 (Adobe) only to adjust brightness, contrast, and sharpness, remove artifacts (i.e., obscuring bubbles), and make composite plates.

**Figure 1.**
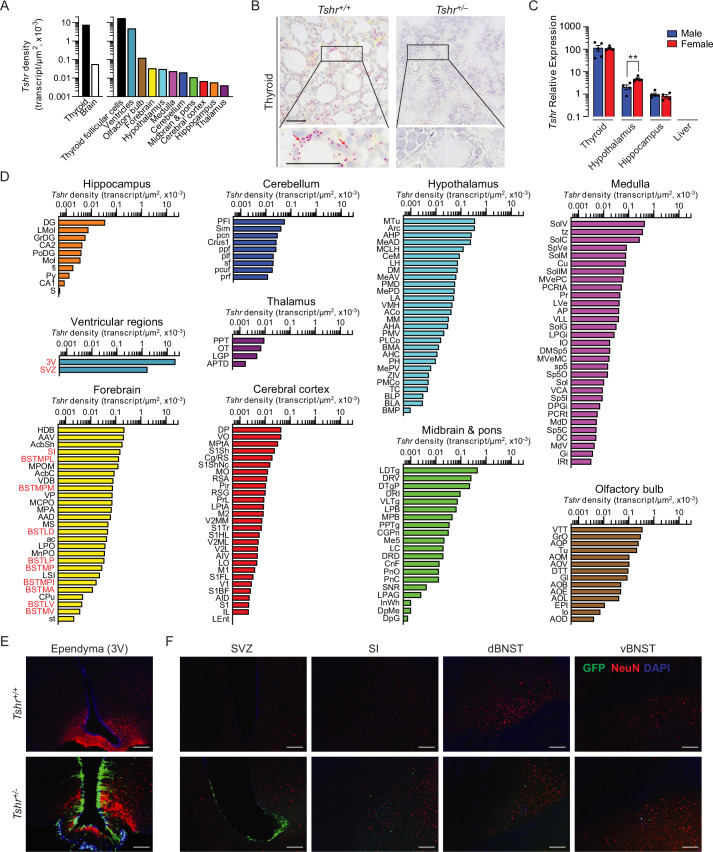
*Tshr* expression in the mouse brain. (**A**) *Tshr* transcript density in the thyroid and various brain regions detected by RNAscope. (**B**) RNAscope probe specificity is confirmed in the *Tshr*^+/+^ thyroid. *Tshr*^–/–^ thyroid was used as negative control. Scale bar: 50 µm. (**C**) *Tshr* expression in the mouse hypothalamus and hippocampus using quantitative PCR. The thyroid and liver serve as positive and negative controls, respectively. Statistics: mean ± SEM, N = 4–5 mice/group, **p<0.01. Data were analyzed by two-tailed Student’s *t*-test using Prism v.9.3.1 (GraphPad, San Diego, CA). Significance was set at p<0.05. (**D**) *Tshr* transcript density in nuclei and subnuclei of the ventricular regions, olfactory bulb, forebrain, hypothalamus, medulla, cerebellum, midbrain and pons, cerebral cortex, hippocampus, and thalamus. (**E**) Abundant GFP immunofluorescence (green) was detected in the ependymal layer of the third ventricle in *Tshr*^+/–^ heterozygous mice, in which a GFP cassette replaced exon 1 of the *Tshr* gene. This GFP signal was absent in *Tshr*^+/+^ mice. (**F**) GFP immunofluorescence was also detected in the subventricular zone (SVZ) of the lateral ventricle, and substantia innominata (SI) and dorsal and ventral bed nucleus of stria terminalis (BNST) in the forebrain of the *Tshr*^+/–^ mice. Sections were co-stained with DAPI (blue) and a neuronal marker, NeuN (red). Scale bar: 100 µm. Figure 1—source data 1.*Tshr* density in brain regions, nuclei, and subnuclei. Figure 1—source data 2.*Tshr* mRNA expression levels in mouse tissues (qPCR).

*Tshr* was detected bilaterally in 173 brain nuclei and subnuclei, in the following descending order of transcript densities: ventricular region, olfactory bulb, forebrain, hypothalamus, medulla, cerebellum, midbrain and pons, cerebral cortex, hippocampus, and thalamus (Figure 1A, Figure 1—source data 1). Importantly, thyroid glands from *Tshr*^–/–^ mice did not show a signal, proving probe specificity (Figure 1B). *Tshr* expression in pooled brain samples was confirmed by qPCR (Figure 1C, Figure 1—source data 2). The hypothalamus and hippocampus expressed *Tshr*, with hypothalamic expression being considerably higher (p<0.01) in females than in males. Furthermore, within other regions of the brain, highest *Tshr* densities were as follows: ependymal layer of the third ventricle (slightly higher than the thyroid follicular cells); VTT in the olfactory bulb; HDB in the forebrain; MTu in the hypothalamus; SoIV in the medulla; PFI in the cerebellum; LDTg in midbrain and pons; DP in the cerebral cortex; DG in hippocampus; and PPT in the thalamus (Figure 1D, Figure 1—source data 1; see Appendix 1 for nomenclature). Raw transcript counts in each region and representative micrographs are shown in Figure 1—figure supplement 1 (Figure 1—figure supplement 1—source data 1) and Figure 1—figure supplement 2, respectively.

**Figure 1—figure supplement 1.**
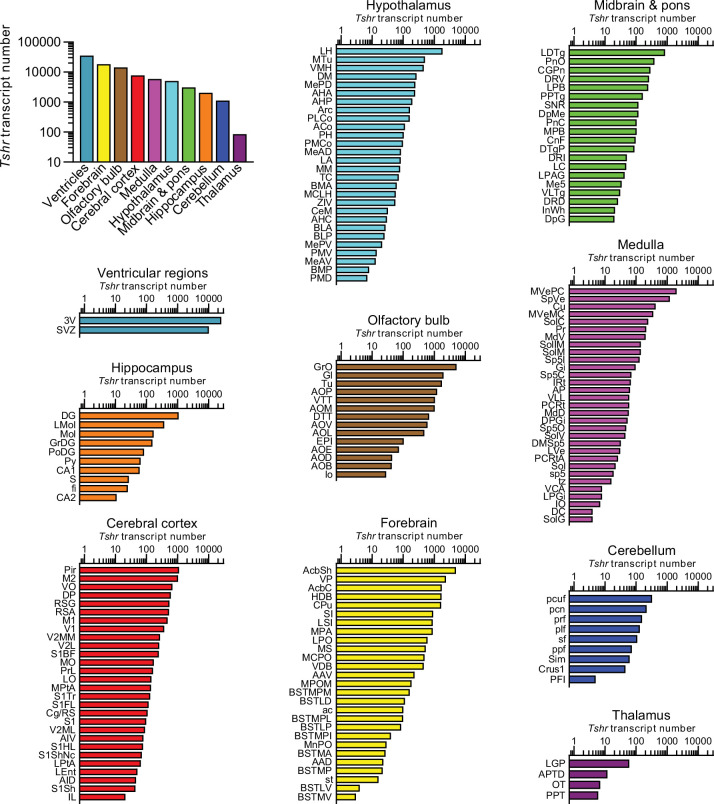
Raw *Tshr* transcript counts in each brain region, nuclei, and subnuclei. Figure 1—figure supplement 1—source data 1.*Tshr* transcript count in brain regions, nuclei, and subnuclei.

**Figure 1—figure supplement 2.**
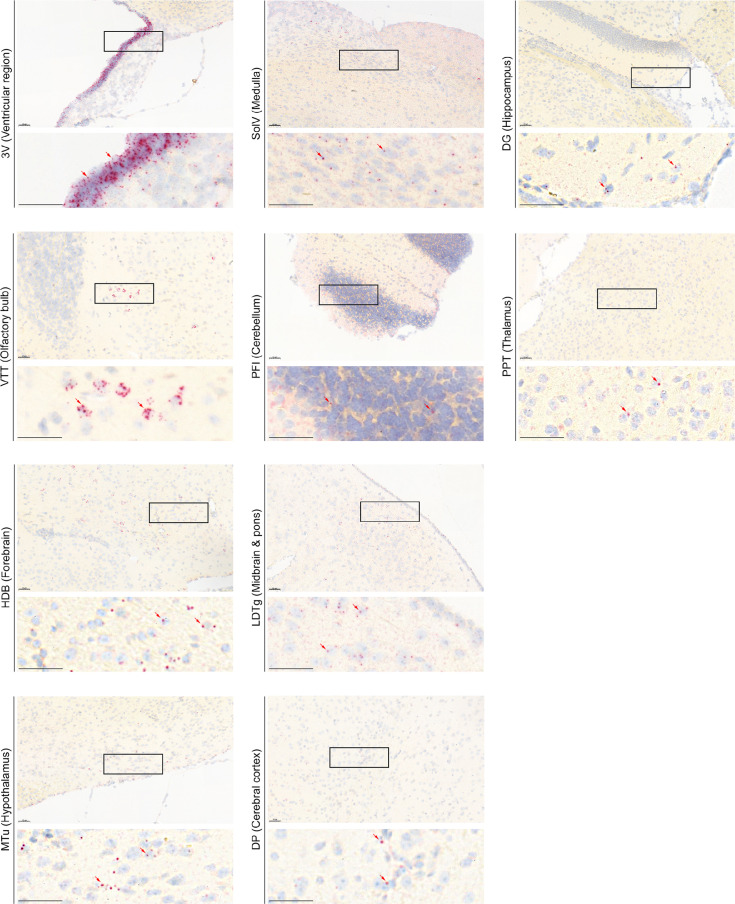
Representative RNAscope micrographs showing *Tshr* transcripts in various regions of the brain. Representative RNAscope micrographs showing *Tshr* transcripts in the ependymal layer of the third ventricle (3V), ventral tenia tecta (VTT) of the olfactory bulb, nucleus of the horizontal limb of the diagonal band (HDB) of the forebrain, medial tuberal nucleus (MTu) of the hypothalamus, solitary nucleus, ventral part (SolV) of the medulla, paraflocculus (PFI) of the cerebellum, laterodorsal tegmental nucleus (LDTg) of the midbrain and pons, dorsal peduncular cortex (DP) of the cerebral cortex, dentate gyrus (DG) of the hippocampus, and posterior pretectal nucleus (PPT) of the thalamus. Scale bar: 50 µm.

For purposes of replicability, we employed a complementary approach to study brain *Tshr* expression—the *Tshr*-deficient mouse—in which exon 1 of the *Tshr* gene is replaced by a *Gfp* cassette. This reporter strategy allows for the in vivo display of *Tshr* locations using GFP immunoreactivity (GFP-ir) as a surrogate for *Tshr* expression (Abe et al., 2003). Of note is that the *Tshr*^+/–^ (haploinsufficient) mouse has one *Tshr* allele intact with normal thyroid function but expresses GFP *in lieu* of one lost allele. In contrast, the *Tshr^+/+^* mouse does not express GFP-ir because both *Tshr* copies are intact and are therefore our negative control.

Consistent with our RNAscope finding, profound GFP-ir was noted in the ependymal region of the third ventricle, mostly in NeuN-negative cells, but with some neuronal localization (Figure 1E). The SVZ of the lateral ventricles, and the SI, and dorsal and ventral BNST of the forebrain also showed GFP-ir, but immunoreactivity was much lower than the ependymal layer of the third ventricle (Figure 1F). In all, while there was overall concordance between the two methodologies for high *Tshr*-expressing areas, GFP-ir was not detected in a number of *Tshr*-positive regions. This latter discrepancy most likely reflects the grossly lower sensitivity of immunohistochemical detection.

**Figure 2.**
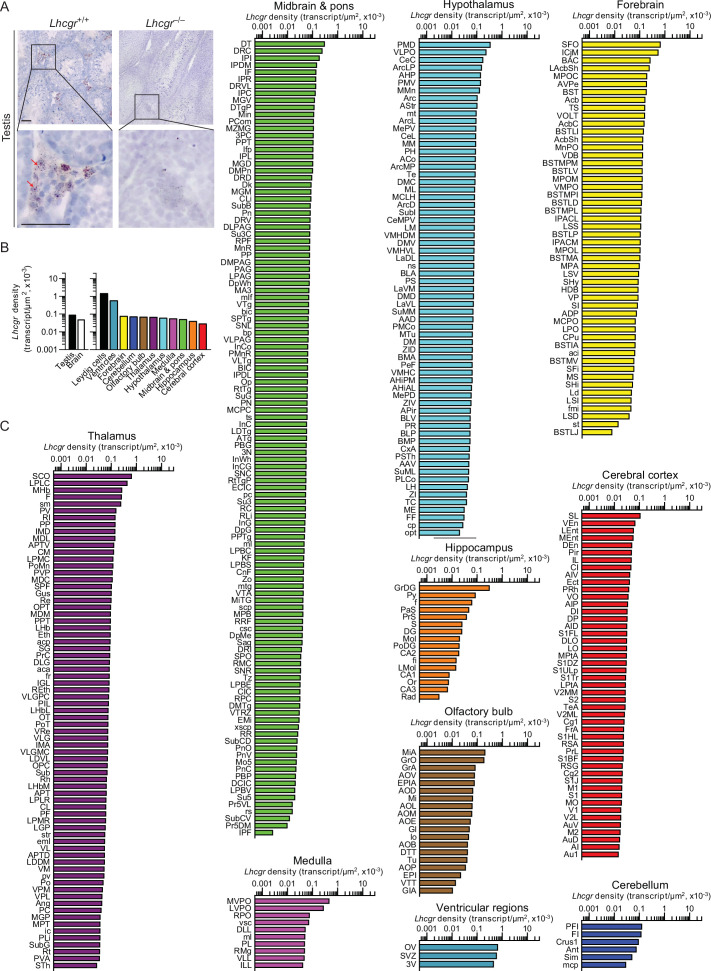
*Lhcgr* expression in the mouse brain. (**A**) RNAscope signals were detected in the Leydig cells, but not juxtaposed Sertoli cells, in the mouse testis, confirming probe specificity. Scale bar: 25 µm. (**B**) *Lhcgr* transcript density in the testis and various brain regions detected by RNAscope. (**C**) *Lhcgr* transcript density in nuclei and subnuclei of the ventricular regions, forebrain, cerebellum, olfactory bulb, thalamus, hypothalamus, medulla, midbrain and pons, hippocampus and cerebral cortex. Figure 2—source data 1.*Lhcgr* density in brain regions, nuclei, and subnuclei.

There is evidence that high LH levels in postmenopausal women correlate with a higher incidence of Alzheimer’s disease (AD) (Henderson et al., 1994; Rocca et al., 2007); LHβ transgenic mice are cognitively impaired Casadesus et al., 2007; LH receptors (LHCGR) are present in the hippocampus (Rao, 2017; Liu et al., 2007); and hCG induces cognitive deficits in rodents (Berry et al., 2008; Barron et al., 2010). Thus, we mapped *Lhcgr* in mouse brain to document expression in 401 brain nuclei and subnuclei. Probe specificity was established by a positive signal in testicular Leydig cells, and with an absent signal in juxtaposed Sertoli cells (Figure 2A). Notably similar to *Tshr* transcripts, the ventricular regions displayed the highest transcript density (Figure 2B, Figure 2—source data 1). Among the brain divisions, the densities were as follows: OV in the ventricular region; SFO in the forebrain; PFI in the cerebellum; MiA in the olfactory bulb; SCO in the thalamus; PMD in the hypothalamus; MVPO in the medulla; DT in midbrain and pons; GrDG in the hippocampus; and SL in the cerebral cortex (Figure 2C, Figure 2—source data 1). Raw transcript counts in each region and representative micrographs are shown in Figure 2—figure supplement 1 (Figure 1—figure supplement 1—source data 1) and Figure 2—figure supplement 2, respectively.

**Figure 2—figure supplement 1.**
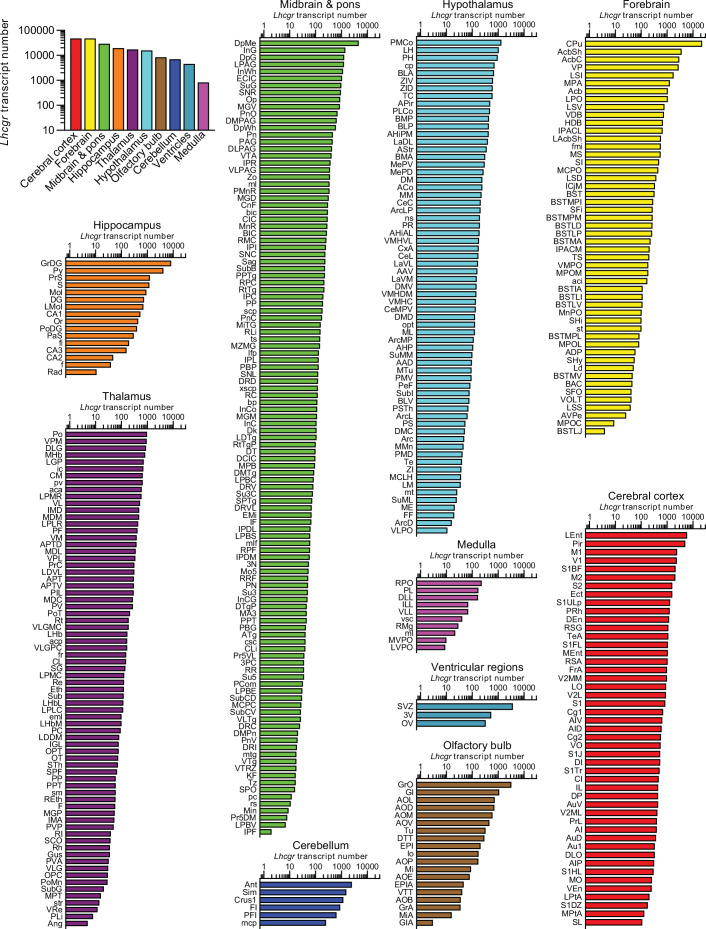
Raw *Lhcgr* transcript counts in each brain region, nuclei, and subnuclei. Figure 2—figure supplement 1—source data 1.*Lhcgr* transcript count in brain regions, nuclei, and subnuclei.

**Figure 2—figure supplement 2.**
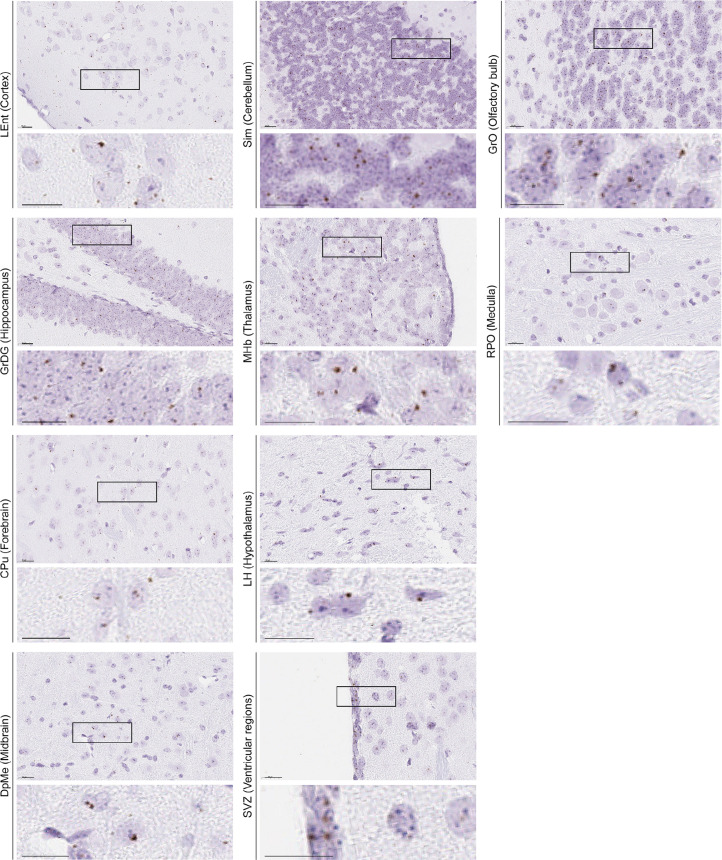
Representative RNAscope micrographs showing *Lhcgr* transcripts in various regions of the brain. Representative RNAscope micrographs showing *Lhcgr* transcripts in the olfactory ventricle (OV), subfornical organ (SFO) of the forebrain, paraflocculus (PFI) of the cerebellum, mitral cell layer of the accessory olfactory bulb (MiA), subcommissural organ (SCO) of the thalamus, premammillary nucleus, dorsal part (PMD) of the hypothalamus, medioventral periolivary nucleus (MVPO) of the medulla, dorsal terminal nucleus of the accessory optic tract (DT) of the midbrain and pons, granular layer of the dentate gyrus (GrDG) of the hippocampus, and semilunar nucleus (SL) of the cerebral cortex. Scale bar: 50 µm.

**Figure 3.**
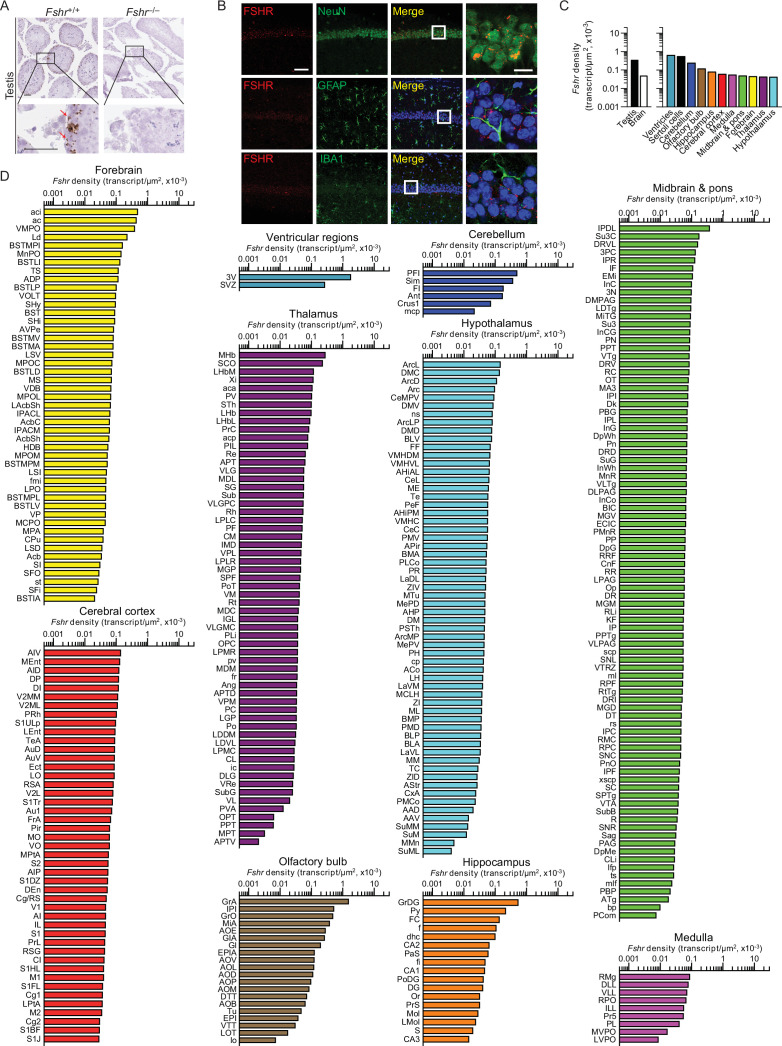
*Fshr* expression in the mouse brain. (**A**) RNAscope signals were detected in the Sertoli cells, but not juxtaposed Leydig cells, in the mouse testis, confirming probe specificity. Scale bar: 50 µm. (**B**) Follicle-stimulating hormone receptor (FSHR) immunofluorescence (red) was colocalized with NeuN-positive neurons, but not with GFAP-positive glial cells or IBA1-positive microglia. Scale bar: 100 µm (magnified view, 10 µm). (**C**) *Fshr* transcript density in the testis and various brain regions detected by RNAscope. (**D**) *Fshr* transcript density in nuclei and subnuclei of the ventricular regions, cerebellum, olfactory bulb, hippocampus, cerebral cortex, medulla, midbrain and pons, forebrain, thalamus, and hypothalamus. Figure 3—source data 1.*Fshr* density in brain regions, nuclei and subnuclei.

We recently reported the expression of FSHR in mouse, rat, and human brains, particularly in AD-vulnerable regions, including hippocampus and cortex (Xiong et al., 2022). We also found that FSH exacerbated AD-like neuropathology and cognitive decline in *3xTg*, *APP*/*PS1,* and *APP*-KI mice, while the inhibition of FSH action rescued this phenotype. Most notably, shRNA-mediated knockdown of the *Fshr* in the hippocampus prevented the onset of AD-like features (Xiong et al., 2022). Here, using RNAscope, we report the expression of *Fshr* at the single-transcript resolution in 353 brain nuclei and subnuclei—and suggest that FSHR in the brain may have roles beyond cognition. Probe specificity was established by a positive signal in testicular Sertoli cells, and an absent signal in juxtaposed Leydig cells and in the testes of *Fshr*^–/–^ mice—as negative controls (Figure 3A). Immunofluorescence confirmed the expression of FSHR in NeuN-positive neurons, but not in GFAP-positive glial cells or IBA1-positive microglia (Figure 3B).

**Figure 3—figure supplement 1.**
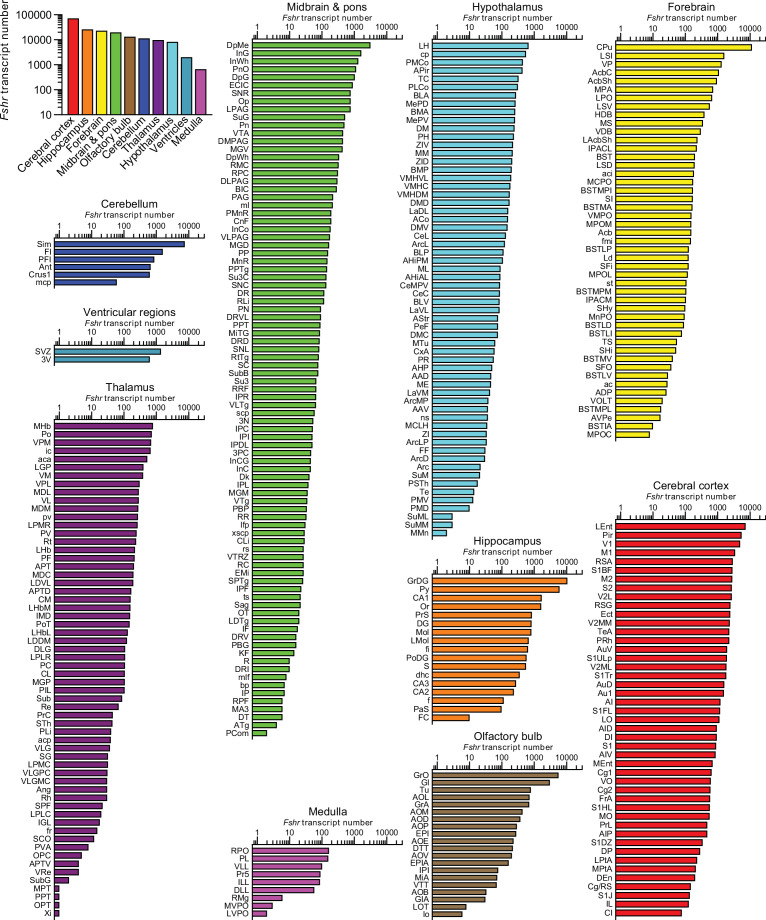
Raw *Fshr* transcript counts in each brain region, nuclei, and subnuclei. Figure 3—figure supplement 1—source data 1.*Fshr* transcript count in brain regions, nuclei, and subnuclei.

**Figure 3—figure supplement 2.**
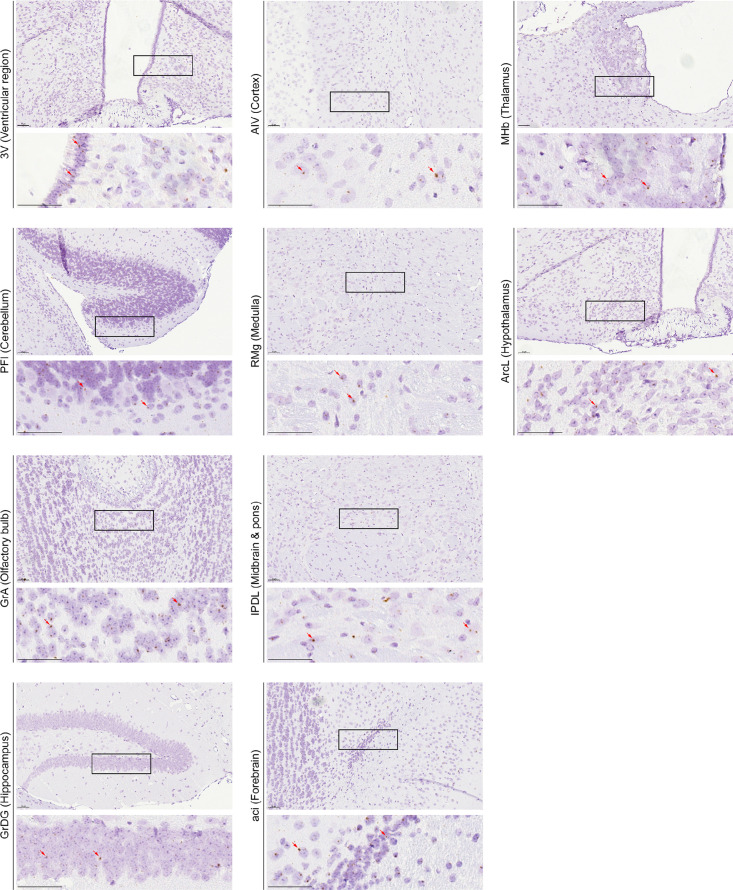
Representative RNAscope micrographs showing *Fshr* transcripts in various regions of the brain. Representative RNAscope micrographs showing *Fshr* transcripts in the ependymal layer of the third ventricle (3V), paraflocculus (PFI) of the cerebellum, granule cell layer of the accessory olfactory bulb (GrA), granular layer of the dentate gyrus (GrDG) of the hippocampus, agranular insular cortex, ventral part (AIV) of the cerebral cortex, raphe magnus nucleus (RMg) of the medulla, interpeduncular nucleus, dorsolateral subnucleus (IPDL) of the midbrain and pons, anterior commissure, intrabulbar part (aci) of the forebrain, medial habenular nucleus (MHb) of the thalamus, and arcuate hypothalamic nucleus, lateral part (ArcL) of the hypothalamus. Scale bar: 50 µm.

*Fshr* transcript density was highest in the ventricular region, followed, in descending order, by the cerebellum, olfactory bulb, hippocampus, cerebral cortex, medulla, midbrain and pons, forebrain, thalamus, and hypothalamus (Figure 3C, Figure 3—source data 1). Within each region, respectively, the highest transcript densities were as follows: ependymal layer of the third ventricle (slightly higher than the testicular Sertoli cells); PFI in the cerebellum; GrA in the olfactory bulb; GrDG in the hippocampus; AIV in the cerebral cortex; RMg in the medulla; MHb in the thalamus; IPDL in midbrain and pons; aci in the forebrain; and ArcL in the hypothalamus (Figure 3D, Figure 3—source data 1). Raw transcript counts in each region and representative micrographs are shown in Figure 3—figure supplement 1 (Figure 3—figure supplement 1—source data 1) and Figure 3—figure supplement 2, respectively.

**Figure 4.**
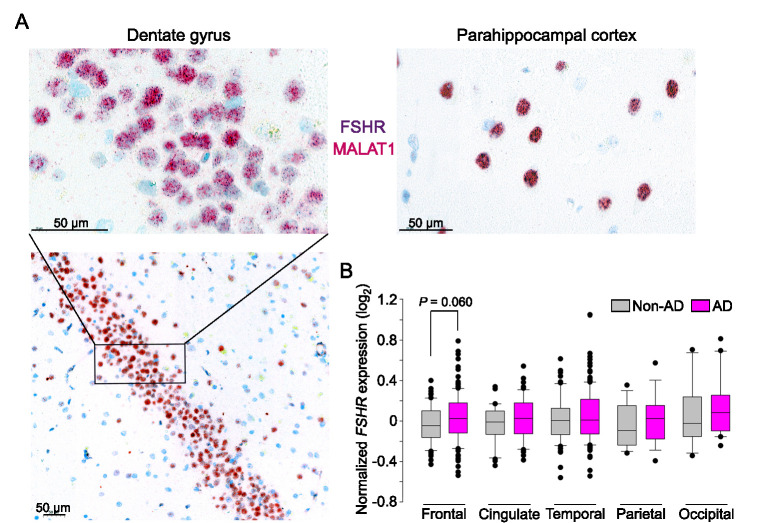
*FSHR* expression in the human brain. (**A**) *FSHR* expression in the human hippocampus and parahippocampal cortex was detected by ViewRNA in neuronal cells that coexpress the noncoding RNA *MALAT1*. (**B**) *FSHR* mRNA expression in the frontal, cingulate, temporal, parietal, and occipital subregions of human cortex in postmortem normal and Alzheimer’s disease (AD) brains (Affymetrix microarray, from GEO accession: GSE84422). Statistics: mean ± SEM, N = 2–15group, Data were analyzed by two-tailed Student’s *t*-test using Prism v.9.3.1 (GraphPad, San Diego, CA). Figure 4—source data 1.*FSHR* mRNA expression in the frontal, cingulate, temporal, parietal, and occipital subregions of human cortex in postmortem normal and Alzheimer’s disease (AD) brains.

We used ViewRNA to examine the expression of *FSHR* transcripts in specific regions of the human brain (Figure 4A). Expression was noted in neuronal cells co-expressing the noncoding RNA *MALAT1* in the GrDG—consistent with the RNAscope data in mouse brain—and in the parahippocampal cortex. This latter data is consistent with *FSHR* expression in a population of excitatory glutamatergic neurons noted in human brain by 10× single-cell RNA-seq (Allen Brain Atlas). Affymetrix microarray analysis confirmed *FSHR* expression in the frontal, cingulate, temporal, parietal, and occipital subregions of human cortex in postmortem normal and AD brains (Figure 4B, Figure 4—source data 1). Interestingly, *FSHR* expression trended to be higher in the frontal cortex of the AD brains compared to that of unaffected brains (p=0.060). In all, the data suggest that, beyond a primary role in regulating cognition, brain FSHR may have a wider role in the central regulation.

## Discussion

The past decade has witnessed the unraveling of nontraditional physiological actions of anterior pituitary glycoprotein hormones, and hence, the unmasking of functional receptors in bone, fat, brain, and immune cells, among other organs (Zaidi et al., 2018; Sun et al., 2006; Liu et al., 2017; Liu et al., 2015; Williams, 2011; Sun et al., 2020; Fields and Shemesh, 2004). We report here for the first time that *Tshr*, *Lhcgr,* and *Fshr* are expressed in multiple brain regions. The data provide new insights into the distributed central neural network of anterior pituitary hormone receptors, particularly in relation to their role in regulating the somatic tissue function. Specifically, we find a surprising and striking overlap in central neural distribution of the three receptors—with highest transcript densities in the ventricular regions. Furthermore, at least for the TSHR and FSHR, expression levels in ependymal layer of the third ventricle was similar to that of the thyroid follicular cells and testicular Sertoli cells, respectively. *Albeit* intriguing, this may suggest a primary role for these receptors in central neural regulation.

Among 173 *Tshr*-positive brain regions, subregions, and nuclei, the ependymal layer of the third ventricle displayed the highest *Tshr* transcript number and density. This region is juxtaposed to the anterior pituitary that produces TSH in response to hypothalamic TRH. Furthermore, TSH has been reported to be expressed in the hypothalamus (DeVito et al., 1986; Hojvat et al., 1983). It is therefore possible that a yet uncharacterized central TSH–TSHR feedback circuit may directly regulate the hypothalamic–pituitary–thyroid axis, thought solely to be controlled by thyroid hormones. To add to this complexity, thyroxine-to-triiodothyronine conversion occurs in tanycytes (Fonseca et al., 2013), which calls into question whether central TSH actions regulate thyroid hormone metabolism in these cells and/or directly modulate hypothalamic TRH neuronal projections. Interestingly, it has been shown that *Tshr* expression is not different between young and old mice (Kerp et al., 2019). However, there is conflicting evidence for the expression of TSH with age—with evidence of no difference between 6-, 15-, and 22-month-old mice (Wang et al., 2019), but a 44% increase in the old rat compared with the young rat (Miler et al., 2019).

The forebrain and olfactory bulb also displayed abundant *Tshr* transcripts, with the highest density in the nucleus of the horizontal limb of the diagonal band (HDB) of the forebrain and ventral tenia tecta (VTT) of the olfactory bulb. These regions are involved, respectively, in learning and odor processing (Shiotani et al., 2020; Cleland and Linster, 2019; McNamara et al., 2004; Chaves-Coira et al., 2018; Zhan et al., 2013). In the hypothalamus, the highest density was found in medial tuberal nucleus (MTu), which controls ingestive behaviors and metabolism (Luo et al., 2018). Finally, we found more recently that the modulation of TSHRs in the bed nucleus of the stria terminalis (BNST), which receives direct afferents from the MTu (Dong and Swanson, 2006), influences anxiety responses, suggesting that TSHR signaling might, in fact, mediate psychosocial behaviors.

While LH has a key role in reproduction and sexual development, we found 401 brain regions, subregions, and nuclei expressing *Lhcgr*. There were nominal differences in *Lhcgr* expression in many brain regions, but the ventricles stood out as having the highest *Lhcgr* density. Two regions deserve special mention. The *Lhcgr*–rich mitral cell layer of the accessory olfactory bulb (MiA) has a known role in scent communication during mating (Gildersleeve et al., 2012; Lydell and Doty, 1972; Huck and Banks, 1984; Singh and Bronstad, 2001). A growing body of evidence suggests that men are attracted to cues of impending ovulation in women, raising an intriguing question on whether cycling hormones affect men’s attraction and sexual behavior (Gildersleeve et al., 2012; Singh and Bronstad, 2001). The broader question is whether LH surges in women during cycling may, in fact, alter male sexual behavior through central mechanisms. Second, a high *Lhcgr* density in the subfornical organ (SFO) of the forebrain was surprising. SFO sends efferent projections to the organum vasculosum of the lamina terminalis (OVLT) (Miselis, 1981; Lind, 1986), which is surrounded by GnRH neurons and contains estrogen receptors (ESR) (Low, 2016). We therefore speculate that circumventricular interactions between LHCGR, LH, GnRH, and ESR underpin the central regulation of reproduction.

RNAscope revealed 353 *Fshr*-expressing brain regions, subregions, and nuclei. Highest expression was noted in the ependymal layer, not surprisingly given its anatomical proximity to the anterior pituitary gland where FSH is produced in response to hypothalamic gonadotropin-releasing hormone (GnRH). The functional significance of *Fshr* expressed in the cerebellum, particularly in the paraflocculus (PFI), is yet unknown. However, other *Fshr*-high subregions, including the granular cell layer of the accessory olfactory bulb (GrA), granular layer of the dentate gyrus (GrDG), and agranular insular cortex (AIV), have known associations with odor processing, learning, memory formation, and anticipation of reward (Eichenbaum, 2001; Nagayama et al., 2014; Kesner and Gilbert, 2007). It is possible that the anosmia of Kallman syndrome, with unclear etiology, may arise from a dysfunctional FSHR-olfaction circuitry. We also find that inactivation of the hippocampal *Fshr* blunts the cognitive impairment and AD-like neuropathology induced by ovariectomy in *3xTg* mice. This data, together with gain- and loss-of-function studies, suggests that hippocampal and cortical FSHR could represent therapeutic targets for AD.

In all, our results provide compelling evidence for multiple central nodes being targets of the anterior pituitary glycoprotein hormones—a paradigm shift that does not conform with the dogma that pituitary hormones are solely master regulators of single bodily processes. Through the intercession of emerging technologies, we compiled the most complete atlas of glycoprotein hormone receptor distribution in the brain at a single-transcript resolution. In addition, we have identified brain sites with the highest transcript expression and density, findings that are imperative toward a better understanding of the neuroanatomical and functional basis of pituitary hormone signaling in the brain. This understanding should provide the foundation for innovative pharmacological interventions for a range of human diseases, wherein direct actions of pituitary hormones have been implicated, importantly, AD.

## Methods

### Mice

We used *Tshr*^+/-^ (strain #004858, Jackson Laboratory), *Lhcgr*^-/-^ (strain #027102, Jackson Laboratory), *Fshr*^-/-^ mice (Dierich et al., 1998), and their wild-type littermates in this study. Adult male mice (~3–4-month-old) were housed in a 12 hr:12 hr light:dark cycle at 22 ± 2°C with ad libitum access to water and regular chow. All procedures were approved by the Mount Sinai Institutional Animal Care and Use Committee (approval number IACUC-2018-0047) and are in accordance with Public Health Service and United States Department of Agriculture guidelines.

### RNAscope

Mouse brain tissue was collected for RNAscope. Briefly, mice were anesthetized with isoflurane (2—3% in oxygen; Baxter Healthcare, Deerfield, IL) and transcardially perfused with 0.9% heparinized saline followed by 4% paraformaldehyde (PFA). Brains were extracted and post-fixed in 4% PFA for 24 hr, dehydrated, and embedded into paraffin. Coronal sections were cut at 5 μm, with every tenth section mounted onto ~20 slides with 2–6 sections on each slide. This method allowed to cover the entire brain and eliminate the likelihood of counting the same transcript twice. Sections were air-dried overnight at room temperature and stored at 4°C until required.

Simultaneous detection of mouse *Tshr*, *Lhcgr,* and *Fshr* was performed on paraffin sections using RNAscope 2.5 LS Multiplex Reagent Kit and RNAscope 2.5 LS Probes, namely, Mm-TSHR, Mm-LHCGR, and Mm-FSHR (Advanced Cell Diagnostics, ACD). RNAscope assays on thyroid glands and testes (positive controls for *Tshr* and *Lhcgr*/*Fshr*, respectively), as well as brains from knockout mice (negative controls), were performed in parallel.

Slides were baked at 60°C for 1 hr, deparaffinized, incubated with hydrogen peroxide for 10 min at room temperature, pretreated with Target Retrieval Reagent for 20 min at 100°C and with Protease III for 30 min at 40°C. Probe hybridization and signal amplification were performed as per the manufacturer’s instructions for chromogenic assays.

Following RNAscope assay, the slides were scanned at ×20 magnification and the digital image analysis was successfully validated using the CaseViewer 2.4 (3DHISTECH) software. The same software was employed to capture and prepare images for the figures in the article. Detection of *Tshr*-, *Lhcgr*-, and *Fshr*-positive cells was also performed using the QuPath-0.2.3 (University of Edinburgh, UK) software based on receptor intensity thresholds, size, and shape.

### Histology and immunofluorescence

Heterozygous *Tshr^+/^*^–^ in which a GFP cassette replaced exon 1 of the *Tshr* gene and their *Tshr^+/+^* littermates were euthanized with carbon dioxide and perfused transcardially with 0.9% heparinized saline followed by 4% PFA in 0.1 M phosphate-buffered saline (PBS; pH 7.4). Brains were collected and post-fixed in the same fixative overnight at 4°C, then transferred to a 30% sucrose solution in 0.1 M PBS with 0.1% sodium azide and stored at 4°C until they were sectioned on a freezing stage sliding microtome at 30 μm. Sections were stored in 0.1 M PBS solution with 0.1% sodium azide until processed for double immunofluorescence.

For the double-label fluorescent immunohistochemistry, free-floating brain sections were rinsed in 0.1 M PBS (2 × 15 min), followed by a 30 min blocking in 3% normal horse serum (Vector Laboratories, Burlingame, CA) and 0.3% Triton X-100 in 0.1 M PBS. Sections were incubated with a mixture of primary rabbit anti-GFP antibody (1:500; Cat# SP3005P, OriGene, Rockville, MD) and mouse anti-NeuN antibody (1:1000; Cat# ab104224, Abcam, Cambridge, MA) for 18 hr. Sections were then incubated with the secondary donkey anti-rabbit Alexa 488 (1:700; Cat# 711-545-152, Jackson ImmunoResearch, West Grove, PA) and donkey anti-mouse DyLight 594 (1:700; Cat# DK-2594, Vector Laboratories) antibodies in 0.1 M PBS for 3 hr at room temperature. For immunohistochemical controls, the primary antibody was either omitted or pre-adsorbed with the immunizing peptide overnight at 4°C, resulting in no immunoreactive staining. In addition, we expectedly did not detect GFP immunoreactivity (-ir) in the *Tshr^+/+^* littermates as the *Tshr* gene was intact and did not express GFP. Sections were mounted onto slides (Superfrost Plus) and cover-slipped using ProLong Gold Antifade Reagent (Life Technologies, Grand Island, NY). All steps were performed at room temperature.

For immunofluorescence staining for FSHR, free-floating brain sections were incubated overnight at 4°C with primary anti-FSHR (1:200; Cat# PA5-50963, Thermo Fisher), anti-NeuN (1:300; Cat# MAB377, Sigma-Aldrich), anti-GFAP (1:400; Cat# MAB360, Sigma-Aldrich), or anti-IBA1 (1:500; Cat# PA5-18039, Thermo Fisher) antibodies. After washing with Tris-buffered saline, the sections were incubated with a mixture of labeled secondary antibodies for detection. DAPI (Sigma-Aldrich) was used for staining nuclei.

### Microarray analysis

Affymetrix Human Genome U133 Plus 2.0 Array data for *FSHR* expression in the frontal, cingulate, temporal, parietal, and occipital cortex from both AD and non-AD human brains were curated from a previously published dataset (GEO accession #GSE84422; Wang et al., 2016).

### Quantitative PCR

For quantitative RT-PCR performed on homogenates of brain tissues, total RNA from the hypothalamus and the hippocampus isolated from five *Tshr^+/+^* mice was extracted using an RNeasy Mini kit (QIAGEN) as per the manufacturer’s protocol. Thyroid and liver tissues were used as positive and negative controls, respectively. RNA was treated with DNAse I (Invitrogen), and reverse-transcribed using the SuperScript II Reverse Transcriptase (Thermo Fisher Scientific). qPCR was performed with a QuantStudio 7 Real-Time PCR system (Applied Biosystems). PCR reaction mix consisted of first-strand cDNA template, exon-spanning primer pairs, and SYBR Green PCR master mix (Thermo Fisher Scientific). Expression of the selected targets was compared to that of a panel of normalizing genes (*Rps11*, *Tubg1,* and *Gapdh*) measured on the same sample in parallel on the same plate, giving a Ct difference (ΔCt) for the normalizing gene minus the test gene. Relative expression levels were calculated by 2^-ΔΔCt^ using thyroid as the reference tissue.

### Quantitation, validation, and statistical analysis

Immunofluorescent images were viewed and captured using ×10 or ×20 objectives with an Observer.Z1 fluorescence microscope (Carl Zeiss, Germany) with appropriate filters for Alexa 488, Cy3, and DAPI. The captured GFP and NeuN images were evaluated and overlaid using AxioVision v.4.8 software (Carl Zeiss, Germany) and ImageJ (NIH, Bethesda, MD).

Data were analyzed by two-tailed Student’s *t*-test using Prism v.9.3.1 (GraphPad, San Diego, CA). Significance was set at p<0.05.

## Data Availability

All data generated or analyzed during this study are included in the manuscript and supporting file.

## Additional files

MDAR checklist

## Appendix 1

Glossary of the brain regions, nuclei, and subnuclei.

**Cerebellum**

Ant anterior lobe cerebellum

Crus1 crus 1 of the ansiform lobule

FI flocculus

mcp middle cerebellar peduncle

pcn precentral fissure

pcuf preculminate fissure

PFI paraflocculus

plf posterolateral fissure

ppf prepyramidal fissure

prf primary fissure

sf secondary fissure

Sim simple lobule

**Cerebral cortex**

AI agranular insular cortex

AID agranular insular cortex, dorsal part

AIP agranular insular cortex, posterior part

AIV agranular insular cortex, ventral part

Au1 primary auditory cortex

AuD secondary auditory cortex, dorsal area

AuV secondary auditory cortex, ventral area

Cg/RS cingular/retrosplenial cortex

Cg1 cingulate cortex, area 1

Cg2 cingulate cortex, area 2

CI caudal interstitial nucleus of the medial longitudinal fasciculus

DEn dorsal endopiriform nucleus

DI dysgranular insular cortex

DLO dorsolateral orbital cortex

DP dorsal peduncular cortex

Ect ectorhinal cortex

FrA frontal association cortex

IL infralimbic cortex

LEnt lateral entorhinal cortex

LO lateral orbital cortex

LPtA lateral parietal association cortex

M1 primary motor cortex

M2 secondary motor cortex

MEnt medial entorhinal cortex

MO medial orbital cortex

MPtA medial parietal association cortex

Pir piriform cortex

PRh perirhinal cortex

PrL prelimbic cortex

RSA retrosplenial agranular cortex

RSG retrosplenial granular cortex

S1 primary somatosensory cortex

S1BF primary somatosensory cortex, barrel field

S1DZ primary somatosensory cortex, dysgranular region

S1FL primary somatosensory cortex, forelimb region

S1HL primary somatosensory cortex, hindlimb region

S1J primary somatosensory cortex, jaw region

S1Sh primary somatosensory cortex, shoulder region

S1ShNc primary somatosensory cortex, shoulder/neck region

S1Tr primary somatosensory cortex, trunk region

S1ULp primary somatosensory cortex, upper lip region

S2 secondary somatosensory cortex

SL semilunar nucleus

TeA temporal association cortex

V1 primary visual cortex

V2L secondary visual cortex, lateral area

V2ML secondary visual cortex, mediolateral area

V2MM secondary visual cortex, mediomedial area

VEn ventral endopiriform nucleus

VO ventral orbital cortex

**Forebrain**

AAD anterior amygdaloid area, dorsal part

AAV anterior amygdaloid area, ventral part

ac anterior commissure

Acb accumbens nucleus

AcbC accumbens nucleus, core

AcbSh accumbens nucleus, shell

aci anterior commissure, intrabulbar part

ADP anterodorsal preoptic nucleus

AVPe anteroventral periventricular nucleus

BAC bed nucleus of the anterior commissure

BST bed nucleus of the stria terminalis

BSTIA bed nucleus of the stria terminalis, intraamygdaloid division

BSTLD bed nucleus of the stria terminalis, lateral division, dorsal part

BSTLI bed nucleus of the stria terminalis, lateral division, intermediate part

BSTLJ bed nucleus of the stria terminalis, lateral division, juxtacapsular part

BSTLP bed nucleus of the stria terminalis, lateral division, posterior part

BSTLV bed nucleus of the stria terminalis, lateral division, ventral part

BSTMA bed nucleus of the stria terminalis, medial division, anterior part

BSTMP bed nucleus of the stria terminalis, medial division, posterior part

BSTMPI bed nucleus of the stria terminalis, medial division, posterointermediate part

BSTMPL bed nucleus of the stria terminalis, medial division, posterolateral part

BSTMPM bed nucleus of the stria terminalis, medial division, posteromedial part

BSTMV bed nucleus of the stria terminalis, medial division, ventral part

CPu caudate putamen (striatum)

fmi forceps minor of the corpus callosum

HDB nucleus of the horizontal limb of the diagonal band

ICjM islands of Calleja, major island

IPACL interstitial nucleus of the posterior limb of the anterior commissure, lateral part

IPACM interstitial nucleus of the posterior limb of the anterior commissure, medial part

LAcbSh lateral accumbens shell

Ld lambdoid septal zone

LPO lateral preoptic area

LSD lateral septal nucleus, dorsal part

LSI lateral septal nucleus, intermediate part

LSS lateral stripe of the striatum

LSV lateral septal nucleus, ventral part

MCPO magnocellular preoptic nucleus

MnPO median preoptic nucleus

MPA medial preoptic area

MPOC medial preoptic nucleus, central part

MPOL medial preoptic nucleus, lateral part

MPOM medial preoptic nucleus, medial part

MS medial septal nucleus

SFi septofimbrial nucleus

SFO subfornical organ

SHi septohippocampal nucleus

SHy septohypothalamic nucleus

SI substantia innominata

st stria terminalis

TS triangular septal nucleus

VDB nucleus of the vertical limb of the diagonal band

VMPO ventromedial preoptic nucleus

VOLT vascular organ of the lamina terminalis

VP ventral pallidum

**Hippocampus**

CA1 field CA1 of hippocampus

CA2 field CA2 of hippocampus

CA3 field CA3 of hippocampus

DG dentate gyrus

dhc dorsal hippocampal commissure

f fornix

FC fasciola cinereum

fi fimbria of the hippocampus

GrDG granular layer of the dentate gyrus

LMol lacunosum moleculare layer of the hippocampus

Mol molecular layer of the dentate gyrus

Or oriens layer of the hippocampus

PaS parasubiculum

PoDG polymorph layer of the dentate gyrus

PrS presubiculum

Py pyramidal tract

Rad stratum radiatum of the hippocampus

S subiculum

**Hypothalamus**

AAD anterior amygdaloid area, dorsal part

AAV anterior amygdaloid area, ventral part

ACo anterior cortical amygdaloid nucleus

AHA anterior hypothalamic area, anterior part

AHC anterior hypothalamic area, central part

AHiAL amygdalohippocampal area, anterolateral part

AHiPM amygdalohippocampal area, posteromedial part

AHP anterior hypothalamic area, posterior part

APir amygdalopiriform transition area

Arc arcuate hypothalamic nucleus

ArcD arcuate hypothalamic nucleus, dorsal part

ArcL arcuate hypothalamic nucleus, lateral part

ArcLP arcuate hypothalamic nucleus, lateroposterior part

ArcMP arcuate hypothalamic nucleus, medial posterior part

AStr amygdalostriatal transition area

BLA basolateral amygdaloid nucleus, anterior part

BLP basolateral amygdaloid nucleus, posterior part

BLV basolateral amygdaloid nucleus, ventral part

BMA basolateral amygdaloid nucleus, anterior part

BMP basomedial amygdaloid nucleus, posterior part

CeC central amygdaloid nucleus, capsular part

CeL central amygdaloid nucleus, lateral division

CeM central amygdaloid nucleus, medial division

CeMPV central amygdaloid nucleus, medial posteroventral part

cp cerebral peduncle, basal part

CxA cortex-amygdala transition zone

DM dorsomedial hypothalamic nucleus

DMC dorsomedial hypothalamic nucleus, compact part

DMD dorsomedial hypothalamic nucleus, dorsal part

DMV dorsomedial hypothalamic nucleus, ventral part

FF fields of Forel

LA lateroanterior hypothalamic nucleus

LaDL lateral amygdaloid nucleus, dorsolateral part

LaVL lateral amygdaloid nucleus, ventrolateral part

LaVM lateral amygdaloid nucleus, ventromedial part

LH lateral hypothalamic area

LM lateral mammillary nucleus

MCLH magnocellular nucleus of the lateral hypothalamus

ME median eminence

MeAD medial amygdaloid nucleus, anteriodorsal part

MeAV medial amygdaloid nucleus, anteroventral part

MePD medial amygdaloid nucleus, posterodorsal part

MePV medial amygdaloid nucleus, posteroventral part

ML medial mammillary nucleus, lateral part

MM medial mammillary nucleus, medial part

MMn medial mammillary nucleus, median part

mt mammillothalamic tract

MTu medial tuberal nucleus

ns nigrostriatal bundle

opt optic tract

PeF perifornical nucleus

PH posterior hypothalamic area

PLCo posterolateral cortical amygdaloid nucleus

PMCo posteromedial cortical amygdaloid nucleus

PMD premammillary nucleus, dorsal part

PMV premammillary nucleus, ventral part

PR prerubral field

PS parastrial nucleus

PSTh parasubthalamic nucleus

Subl subincertal nucleus

SuM supramammillary nucleus

SuML supramammillary nucleus, lateral part

SuMM supramammillary nucleus, medial part

TC tuber cinereum area

Te terete hypothalamic nucleus

VLPO ventrolateral preoptic nucleus

VMH ventromedial hypothalamic nucleus

VMHC ventromedial hypothalamic nucleus, central part

VMHDM ventromedial hypothalamic nucleus, dorsomedial part

VMHVL ventromedial hypothalamic nucleus, ventrolateral part

ZI zona incerta

ZID zona incerta, dorsal part

ZIV zona incerta, ventral part

**Medulla**

AP area postrema

Cu cuneate nucleus

DC dorsal cochlear nucleus

DLL dorsal nucleus of the lateral lemniscus

DMSp5 dorsomedial spinal trigeminal nucleus

DPGi dorsal paragigantocellular nucleus

Gi gigantocellular reticular nucleus

ILL intermediate nucleus of the lateral lemniscus

IO inferior olive

IRt intermediate reticular nucleus

LPGi lateral paragigantocellular nucleus

LVe lateral vestibular nucleus

LVPO lateroventral periolivary nucleus

MdD medullary reticular nucleus, dorsal part

MdV medullary reticular nucleus, ventral part

ml medial lemniscus

MVeMC medial vestibular nucleus, magnocellular part

MVePC medial vestibular nucleus, parvicellular part

MVPO medioventral periolivary nucleus

PCRt parvicellular reticular nucleus

PCRtA parvicellular reticular nucleus, alpha part

PL paralemniscal nucleus

Pr prepositus nucleus

Pr5 principal sensory trigeminal nucleus

RMg raphe magnus nucleus

RPO rostral periolivary region

Sol solitary tract

SolC nucleus of the solitary tract, commissural part

SolG nucleus of the solitary tract, gelatinous part

SolIM nucleus of the solitary tract, intermediate part

SolM nucleus of the solitary tract, medial part

SolV solitary nucleus, ventral part

sp5 spinal trigeminal tract

Sp5C spinal trigeminal nucleus, caudal part

Sp5I spinal trigeminal nucleus, interpolar part

Sp5O spinal trigeminal nucleus, oral part

SpVe spinal vestibular nucleus

tz trapezoid body

VCA ventral cochlear nucleus, anterior part

VLL ventral nucleus of the lateral lemniscus

vsc ventral spinocerebellar tract

**Midbrain and pons**

3N oculomotor nucleus

3PC oculomotor nucleus, parvicellular part

ATg anterior tegmental nucleus

BIC nucleus of the brachium of the inferior colliculus

bic brachium of the inferior colliculus

bp brachium pontis (stem of middle cerebellar peduncle)

CGPn central gray of the pons

CIC central nucleus of the inferior colliculus

CLi caudal linear nucleus of the raphe

CnF cuneiform nucleus

csc commissure of the superior colliculus

DCIC dorsal cortex of the inferior colliculus

Dk nucleus of Darkschewitsch

DLPAG dorsolateral periaqueductal gray

DMPAG dorsomedial periaqueductal gray

DMPn dorsomedial pontine nucleus

DMTg dorsomedial tegmental area

DpG deep gray layer of the superior colliculus

DpMe deep mesencephalic nucleus

DpWh deep white layer of the superior colliculus

DR dorsal raphe nucleus

DRC dorsal raphe nucleus, caudal part

DRD dorsal raphe nucleus, dorsal part

DRI dorsal raphe nucleus, interfascicular part

DRV dorsal raphe nucleus, ventral part

DRVL dorsal raphe nucleus, ventrolateral part

DT dorsal terminal nucleus of the accessory optic tract

DTgP dorsal tegmental nucleus, pericentral part

ECIC external cortex of the inferior colliculus

EMi epimicrocellular nucleus

IF interfascicular nucleus

InC interstitial nucleus of Cajal

InCG interstitial nucleus of Cajal, greater part

InCo intercollicular nucleus

InG intermediate gray layer of the superior colliculus

InWh intermediate white layer of the superior colliculus

IP interpeduncular nucleus

IPC interpeduncular nucleus, caudal subnucleus

IPDL interpeduncular nucleus, dorsolateral subnucleus

IPDM interpeduncular nucleus, dorsomedial subnucleus

IPF interpeduncular fossa

IPI interpeduncular nucleus, intermediate subnucleus

IPL interpeduncular nucleus, lateral subnucleus

IPR interpeduncular nucleus, rostral subnucleus

KF Kölliker-Fuse nucleus

LC locus coeruleus

LDTg laterodorsal tegmental nucleus

lfp longitudinal fasciculus of the pons

LPAG lateral periaqueductal gray

LPB lateral parabrachial nucleus

LPBC lateral parabrachial nucleus, central part

LPBE lateral parabrachial nucleus, external part

LPBS lateral parabrachial nucleus, superior part

LPBV lateral parabrachial nucleus, ventral part

MA3 medial accessory oculomotor nucleus

MCPC magnocellular nucleus of the posterior commissure

Me5 mesencephalic trigeminal nucleus

MGD medial geniculate nucleus, dorsal part

MGM medial geniculate nucleus, medial part

MGV medial geniculate nucleus, ventral part

Min minimus nucleus

MiTG microcellular tegmental nucleus

ml medial lemniscus

mlf medial longitudinal fasciculus

MnR median raphe nucleus

Mo5 motor trigeminal nucleus

MPB medial parabrachial nucleus

mtg mammillotegmental tract

MZMG marginal zone of the medial geniculate

Op optic nerve layer of the superior colliculus

OT nucleus of the optic tract

PAG periaqueductal gray

PBG parabigeminal nucleus

PBP parabrachial pigmented nucleus

pc posterior commissure

PCom nucleus of the posterior commissure

PMnR paramedian raphe nucleus

Pn pontine nuclei

PN paranigral nucleus

PnC pontine reticular nucleus, caudal part

PnO pontine reticular nucleus, oral part

PnV pontine reticular nucleus, ventral part

PP peripeduncular nucleus

PPT posterior pretectal nucleus

PPTg pedunculopontine tegmental nucleus

Pr5DM principal sensory trigeminal nucleus, dorsomedial part

Pr5VL principal sensory trigeminal nucleus, ventrolateral part

R red nucleus

RC raphe cap

RLi rostral linear nucleus of the raphe

RMC red nucleus, magnocellular part

RPC red nucleus, parvicellular part

RPF retroparafascicular nucleus

RR retrorubral nucleus

RRF retrorubral field

rs rubrospinal tract

RtTg reticulotegmental nucleus of the pons

RtTgP reticulotegmental nucleus of the pons, pericentral part

Sag sagulum nucleus

SC superior colliculus

scp superior cerebellar peduncle (brachium conjunctivum)

SNC substantia nigra, compact part

SNL substantia nigra, lateral part

SNR substantia nigra, reticular part

SPO superior paraolivary nucleus

SPTg subpedencular tegmental nucleus

Su3 supraoculomotor periaqueductal gray

Su3C supraoculomotor cap

Su5 supratrigeminal nucleus

SubB subbrachial nucleus

SubCD subcoeruleus nucleus, dorsal part

SubCV subcoeruleus nucleus, ventral part

SuG superficial gray layer of the superior colliculus

ts tectospinal tract

Tz nucleus of the trapezoid body

VLPAG ventrolateral periaqueductal gray

VLTg ventrolateral tegmental area

VTA ventral tegmental area

VTg ventral tegmental nucleus

VTRZ visual tegmental relay zone

xscp decussation of the superior cerebellar peduncle

Zo zonal layer of the superior colliculus

**Olfactory bulb**

AOB accessory olfactory bulb

AOD anterior olfactory nucleus, dorsal part

AOE anterior olfactory nucleus, external part

AOL anterior olfactory nucleus, lateral part

AOM anterior olfactory nucleus, medial part

AOP anterior olfactory nucleus, posterior part

AOV anterior olfactory nucleus, ventral part

DTT dorsal tenia tecta

EPI external plexiform layer of the olfactory bulb

EPIA external plexiform layer of the accessory olfactory bulb

GIA glomerular layer of the accessory olfactory bulb

Gl glomerular layer of the olfactory bulb

GrA granule cell layer of the accessory olfactory bulb

GrO granular cell layer of the olfactory bulb

IPI interpeduncular nucleus, intermediate subnucleus

lo lateral olfactory tract

LOT nucleus of the lateral olfactory tract

Mi mitral cell layer of the olfactory bulb

MiA mitral cell layer of the accessory olfactory bulb

Tu olfactory tubercle

VTT ventral tenia tecta

**Thalamus**

aca anterior commissure, anterior part

acp anterior commissure, posterior

Ang angular thalamic nucleus

APT anterior pretectal nucleus

APTD anterior pretectal nucleus, dorsal part

APTV anterior pretectal nucleus, ventral part

CL centrolateral thalamic nucleus

CM central medial thalamic nucleus

DLG dorsal lateral geniculate nucleus

eml external medullary lamina

Eth ethmoid thalamic nucleus

F nucleus of the fields of Forel

fr fasciculus retroflexus

Gus gustatory thalamic nucleus

ic internal capsule

IGL intergeniculate leaf

IMA intramedullary thalamic area

IMD intermediodorsal thalamic nucleus

LDDM laterodorsal thalamic nucleus, dorsomedial part

LDVL laterodorsal thalamic nucleus, ventrolateral part

LGP lateral globus pallidus

LHb lateral habenular nucleus

LHbL lateral habenular nucleus, lateral part

LHbM lateral habenular nucleus, medial part

LPLC lateral posterior thalamic nucleus, laterocaudal part

LPLR lateral posterior thalamic nucleus, laterorostral part

LPMC lateral posterior thalamic nucleus, mediocaudal part

LPMR lateral posterior thalamic nucleus, mediorostral part

MDC mediodorsal thalamic nucleus, central part

MDL mediodorsal thalamic nucleus, lateral part

MDM mediodorsal thalamic nucleus, medial part

MGP medial globus pallidus (entopeduncular nucleus)

MHb medial habenular nucleus

MPT medial pretectal nucleus

OPC oval paracentral thalamic nucleus

OPT olivary pretectal nucleus

OT nucleus of the optic tract

PC paracentral thalamic nucleus

PF parafascicular thalamic nucleus

PIL posterior intralaminar thalamic nucleus

PLi posterior limitans thalamic nucleus

Po posterior thalamic nuclear group

PoMn posteromedian thalamic nucleus

PoT posterior thalamic nuclear group, triangular part

PP peripeduncular nucleus

PPT posterior pretectal nucleus

PrC precommissural nucleus

pv periventricular fiber system

PV paraventricular thalamic nucleus

PVA paraventricular thalamic nucleus, anterior part

PVP paraventricular thalamic nucleus, posterior part

Re reuniens thalamic nucleus

REth retroethmoid nucleus

Rh rhomboid thalamic nucleus

RI rostral interstitial nucleus of medial longitudinal fasciculus

Rt reticular thalamic nucleus

SCO subcommissural organ

SG suprageniculate thalamic nucleus

sm stria medullaris of the thalamus

SPF subparafascicular thalamic nucleus

STh subthalamic nucleus

str superior thalamic radiation

Sub submedius thalamic nucleus

SubG subgeniculate nucleus

VL ventrolateral thalamic nucleus

VLG ventral lateral geniculate nucleus

VLGMC ventral lateral geniculate nucleus, magnocellular part

VLGPC ventral lateral geniculate nucleus, parvicellular part

VM ventromedial thalamic nucleus

VPL ventral posterolateral thalamic nucleus

VPM ventral posteromedial thalamic nucleus

VRe ventral reuniens thalamic nucleus

Xi xiphoid thalamic nucleus

**Ventricular zones**

3V 3rd ventricle

OV olfactory ventricle (olfactory part of lateral ventricle)

SVZ subventricular zone of the lateral ventricle
